# Nanoscale Vertical Arrays of Gold Nanorods by Self-Assembly: Physical Mechanism and Application

**DOI:** 10.1186/s11671-019-2946-6

**Published:** 2019-04-02

**Authors:** Jun Dong, Xing Zhao, Wei Gao, Qingyan Han, Jianxia Qi, Yongkai Wang, Sandong Guo, Mengtao Sun

**Affiliations:** 1 School of Electronic Engineering, Xi’an University of Posts and Telecommunications, Xi’an, 710121 China; 2 School of Mathematics and Physics, Beijing Key Laboratory for Magneto-Photoelectrical Composite and Interface Science, Center for Green Innovation, University of Science and Technology Beijing, Beijing, 100083 China

**Keywords:** Gold nanorods, Self-assembled method, Surface-enhanced Raman scattering, Surface plasmon resonance

## Abstract

The unique photonic effect of self-assembled metal nanoparticles is widely used in many applications. In this article, we prepared self-assembled gold nanorod (GNR) vertical arrays substrate by an evaporation method and found that the morphology of the substrate can be effectively regulated by changing the immersion time in the target molecules solution to obtain different Raman enhancement effects. We separately calculated the local electromagnetic field of the GNR vertical arrays and disorder substrate by the finite element method (FEM), which was consistent with the experimental results. Based on optimal soaking time, the sensitivity, reproducibility, and stability of substrates were separately studied. The experimental results show that the GNR vertical arrays can detect Rhodamine 6G (Rh6G) at concentrations as low as 10^−11^ M and exhibit good reproducibility and stability due to local electromagnetic (EM) field enhancement caused by the coupling of adjacent nanorods. Thus, our work can demonstrate that the substrate has excellent surface-enhanced Raman scattering (SERS) activity and the obtained GNR vertical arrays have great potential for biosensor and biodetection.

## Introduction

Noble metal nanostructures (gold, silver, copper, etc.) can generate localized EM fields on their surfaces using visible radiation, which provides favorable conditions for enhancing the spectral signals of the probe molecules [1, 2]. The specific excitation conditions can generate surface plasmon resonance (SPR) on the surface of the metal nanostructures, which have important research significance and novel optical effects in plasmonics, including surface-enhanced fluorescence (SEF) and SERS. Owning to high sensitivity, fast response, and fingerprint effect, SERS has great potential for the applications, such as material detection, biomedicine, and sensors, etc [3–7]. In general, SERS is grouped into two categories “local EM field enhancement” and “chemical enhancement mechanisms” [8]. It is well accepted that “EM field enhancement” plays a major role in SERS and it shows enhancements from 4 to 11 orders of magnitude. The “hot spots” produced between adjacent metal nanoparticles can lead to a huge local EM field near the metallic surface; therefore, the Raman scattering of the molecules located in the EM field can be enhanced. In order to obtain good SERS effect, a well-shaped metal substrate, suitable probe molecules, and selection of excitation conditions are all crucial [9]. In recent years, there have been numerous reports on SERS. Sun et al. prepared silver nanoarrays by template method which possessed excellent SERS effect on the substrate [10]. Lu et al. discovered that silver nanowires can produce surface morphological changes at the focus of the laser and had strong SERS effects on the surrounding target molecules [11]. Cho et al. detected Raman signals of 4-NTP with low concentrations on silver dendrite nanocrystal substrate [12]. Although there have been many reports about SERS, the promotion of SERS still faces many challenges. For example, preparing low-cost, large-area uniform substrate and achieving ultra-sensitive detection, etc.

Self-assembled metal nanostructures as promising substrates have attracted more and more attention in both experimental and theoretical aspects [13–18]. Compared with single nanoparticles, the local EM field of the self-assembled metal nanoparticles shows extremely unique optical property. Moreover, self-assembly substrate has the advantages of low cost, easy handling, and uniform distribution over a large area. Combining these advantages, it can be considered that the self-assembled substrate has great potential in promoting SERS. Recently, some research groups have reported gold nanorod (GNR) self-assembled substrates for SERS [19–21]. However, as far as we know, the influence of a change in the morphology of the GNR vertical arrays substrate on the Raman signals of the target molecules has been rarely studied. Herein, we firstly prepared self-assembled GNR vertical arrays substrate by the evaporation method [22]. And then, the substrate was immersed in a probe molecule solution; the morphology of the GNRs vertical arrays was regulated by changing the soaking time. Finally, the Raman spectra of rhodamine 6G (Rh6G) and crystal violet (CV) on the substrate were obtained under specific excitation conditions. In order to verify the results of the experiment, we used SEM images of the GNR vertical arrays and disorder substrates to simulate the local field distribution of substrates by FEM. The result shows that the simulation calculation is almost consistent with the experimental data. In addition, we also study the detection sensitivity, reproducibility, and stability of the SERS substrate based on the above optimal soaking time and discussed the experimental results. Excellent sensitivity, reproducibility, and stability can indicate that GNR vertical arrays substrate can serve as a good candidate for the application of optical sensor area.

## Methods and Experiment

### Material

Rh6G (laser grade) was purchased from Exciton (America), CV was purchased from Sigma-Aldrich, gold chloride tetrahydrate, ethanol, silver nitrate, and hydrochloric acid were purchased from Sinopharm Chemical Reagent Co., Ltd. (China). Cetyltrimethyl ammonium bromide (CTAB), sodium borohydride, and ascorbic acid are purchased from Shanghai Aladdin Bio-Chem Technology Co., Ltd. (China). Silicon wafers (Si) were purchased from Li Jing Photoelectric Technology Co. Ltd. (Zhejiang, China). All reagents are used without further purification. Deionized water was used throughout the experiment.

### Preparation of GNR Vertical Arrays

**Fig. 1:**
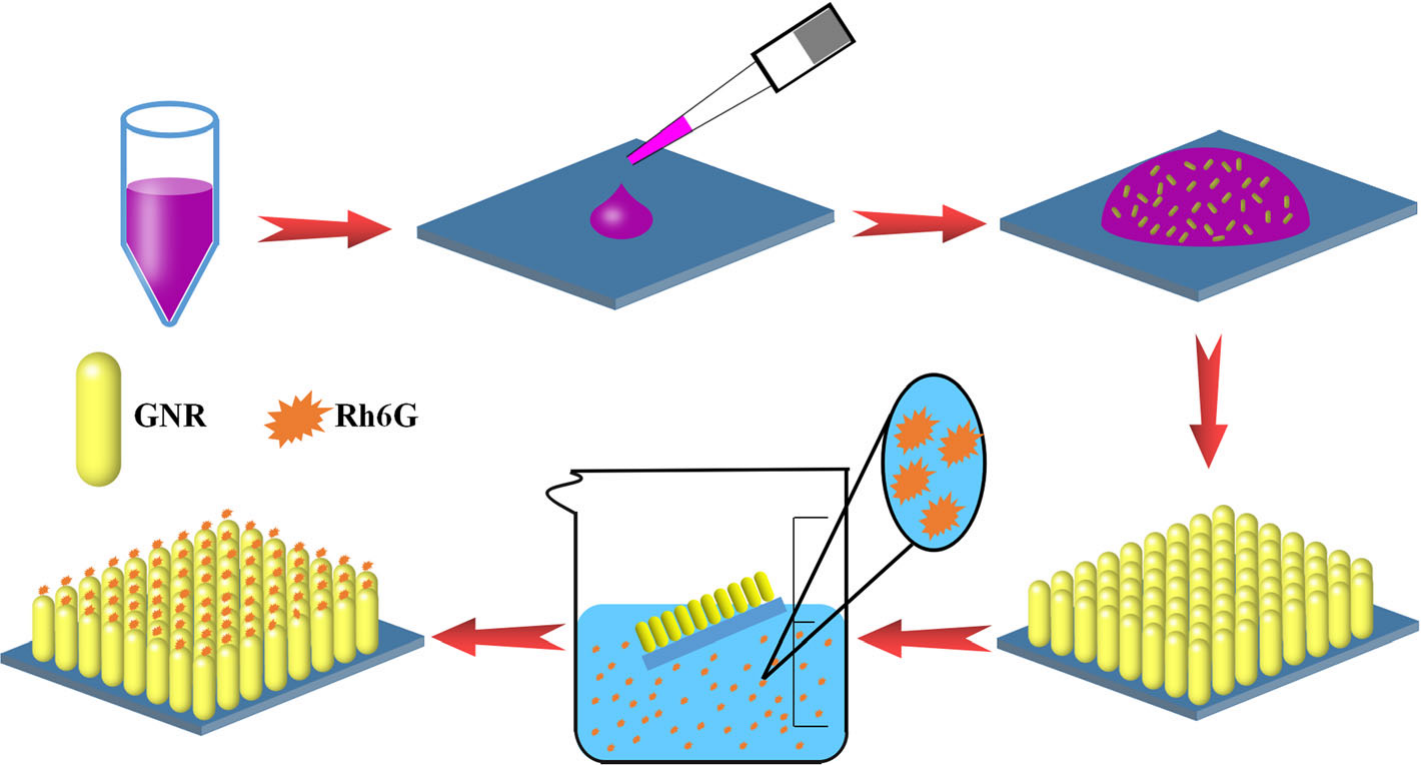
The scheme of the preparation process of GNR vertical arrays

The GNRs were performed via a modified seed-mediated growth method [23, 24]. The obtained GNR solution was centrifuged three times at 10,000 rpm for 5 min to remove excess CTAB. Based on previous methods [22], we utilized solvent evaporation method to get GNR vertical arrays. Then, the substrate was soaked in the solution of the probe molecules. The sample preparation process is shown in Fig. 1. At the end of the process, the substrate was gently pulled out, rinsed with alcohol, and then dried.

### Characterization

The size and morphology of the GNR vertical array were measured with scanning electron microscope (SEM, Nova Nano 450). Raman signals were collected with confocal Raman microscopy (LabRAM HR Evolution, HORIBA Jobin Yvon SAS). The CW laser with 532 nm was used as an excitation source, and the power of laser is 0.5 mW. The samples were exposed to the microscope (× 50), and the integration time was set as 1 s.

## Results and Discussion

### Mechanism of Gold Nanorod Self-Assembly

In general, capillary edgeward flow is generated inside the droplets to carry the suspended GNRs to the edge of the droplets, causing a large number of GNRs deposit at the edge to form a disordered GNR distribution, which is known as the “coffee ring” effect [25, 26]. Nevertheless, GNRs in aqueous solution are arranged side by side to form an initial six deformation structure by attractive forces and electrostatic forces under appropriate conditions. Marangoni flow and contact line receding of droplet cause the free GNRs in solution to accumulate around the initial model, resulting in the area of the GNR vertical arrays to continuously increase. Terminally, the vertical arrays are fixed on the substrate due to gravity and van der Waals interactions. In the process of forming GNR vertical arrays, there are three main influencing factors: van der Waals force, depletion force, and electrostatic force [27]. The van der Waals force and the induced depletion force belong to an attractive force, and electrostatic force belongs to a repulsive force. The van der Waals forces and depletion forces bring adjacent GNRs closely together. Electrostatic repulsive force stabilizes GNRs within a certain distance and prevents them from randomly gathering. The synergy between attractive force and repulsive force induces GNRs into high-ordered arrays.

Temperature and humidity are important influencing factors in self-assembly. GNR droplet forms “coffee ring” in a high temperature or low-humidity environment. In the evaporation process, the contact line of the droplet is pinned. Due to the higher evaporation rate at the edge of the droplet, the GNRs are carried to the pinning contact line by the capillary flow and deposited to form a ring pattern. In contrast, the GNR solution produces Marangoni flow, and GNRs are close-packed and high ordered under the appropriate circumstance. Moreover, the surfactant concentration also plays a key role in the self-assembly process. Many researches have shown that increasing the concentration of surfactant CTAB is beneficial to the formation of GNR vertical arrays substrates [28, 29]. The main reason is that the GNRs are driven by the capillary flow and move around the contact line of the droplet. If the surfactant concentration is too low to form Marangoni flow, a large number of particles will deposit around the contact line to cause disordered distribution. Conversely, increasing the surfactant concentration can result in numerous surfactant molecules to be pushed onto the contact line and more easily produce Marangoni flow. A part of the GNRs are deposited near the contact line during the evaporation process, and excess nanoparticles are returned to the center of the drop under the Marangoni eddy to complete the subsequent assembly. It can be concluded that the nanorods are controlled by the Marangoni flow to complete the GNR ordered arrays. Controlling these influence factors can help to form ordered and large-area GNR vertical arrays, which can provide a reliable support for the subsequent spectrum.

### Morphological of Gold Nanorods and Vertical Array

The preparation process and subsequent operation of the GNR vertical arrays are given in Fig. 1. For simplicity, the experimental procedure is only schematically represented. In brief, 5 μl drops from a centrifuged GNR solution were dripped on a washed silicon wafer with acetone, ethanol, and deionized water (6 × 6 mm^2^ in size). Then, the silicon wafer with GNR droplet was placed in the circumstance of 21 °C and humidity of 85% to slowly evaporate. After 72 h, side by side GNR vertical arrays were obtained. According to previous reports, we utilized the “seed-mediated growth” to synthesize GNRs [23, 24].

**Fig. 2:**
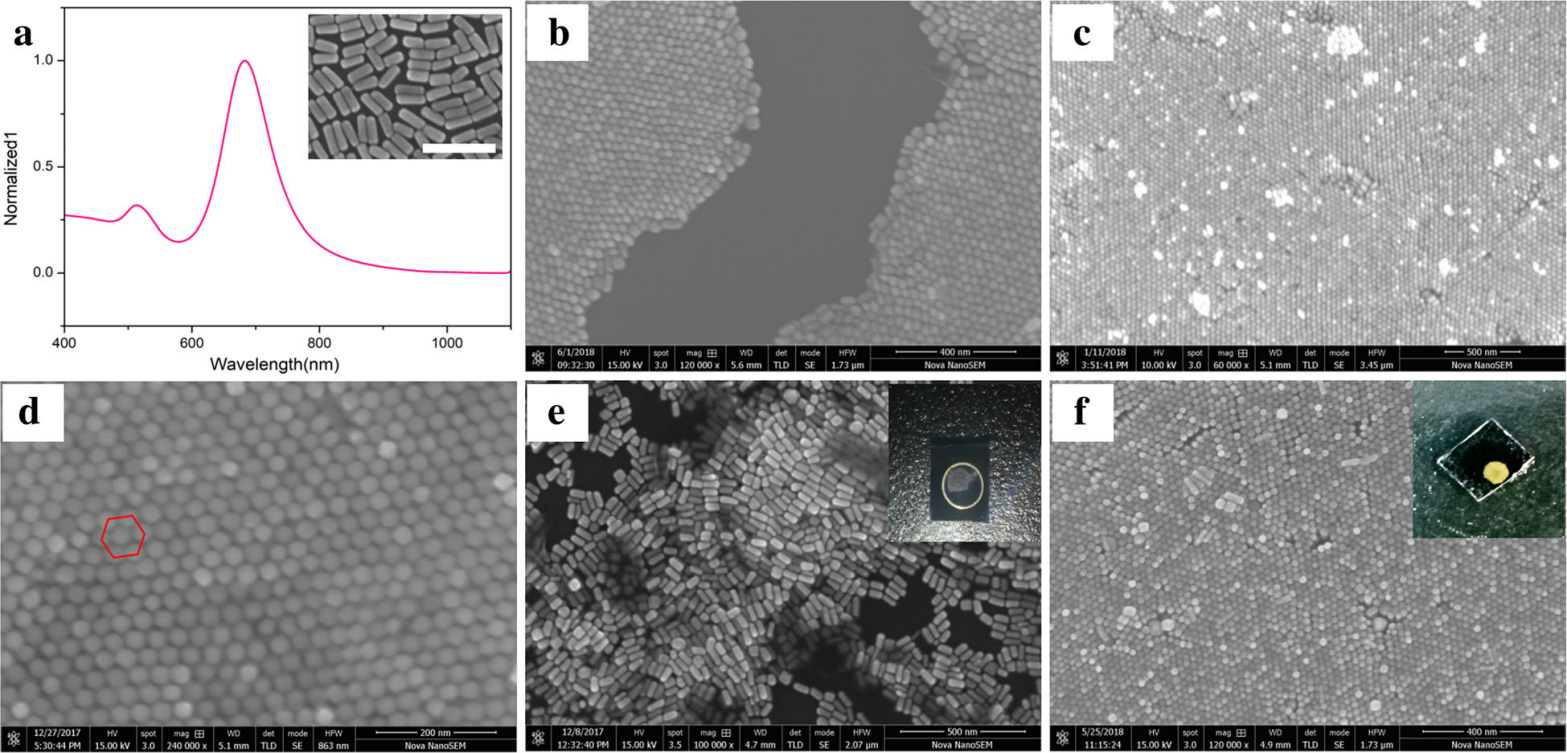
**a** The ultraviolet-visible absorption spectrum of GNR. **b**–**d** The typical SEM images of the GNR vertical arrays. **e**, **f** Correspond to the SEM images of coffee rings and coffee stain samples

Figure 2a shows the normalized ultraviolet-visible absorption spectrum of GNR. The two absorption peaks of the GNR are observed, which are attributed to the longitudinal peak at 690 nm and the transverse peak at 520 nm. Generally speaking, the longitudinal absorption peak corresponding to long GNRs is red-shifted. Within a certain range, the aspect ratio of the GNRs can be adjusted by changing the amount of silver nitrate [23]. The “inset SEM” in the upper right corner of Fig. 2a shows that the GNRs have a good appearance. We use CTAB as a surfactant to prepare GNRs with a length of approximately 69 ± 5 nm, a width of approximately 24 ± 2 nm, and an aspect ratio of approximately 3. Many previous researches have reported that GNRs with a relatively small aspect ratio can promote the formation of vertical arrays [28]. Figure 2b shows a SEM image of vertically self-assembled GNRs monolayer formed on a silicon wafer, and Fig. 2c reveals that the GNRs are successfully self-assembled on the surface of the silicon wafer and have good reproducibility over a large area. The large area array substrate provides favorable conditions for subsequent spectral development. The anisotropy of the GNRs can be clearly observed from Fig. 2d indicating that the GNRs are perpendicular to the surface of the silicon wafer, and the hexagonally close-packed structure is obtained (marked by red lines). The internal gap distance between two adjacent nanorods in vertical arrays is approximately 3 nm, which is assigned to the length of the double-layered cationic surfactant CTAB, and is sufficient to generate “hot spots” [30, 31]. Because of contact line pinning, the GNRs are pushed to the edge of the droplet to form a coffee ring pattern under capillary edgeward flow, as shown in Fig. 2e. However, a large area of GNR vertical arrays can be obtained in the “coffee stain” sample due to contact line receding, as shown in Fig. 2f, which is consistent with previous reports [14, 28].

### Spectrum Enhancement with GNR Vertical Array

**Fig. 3:**
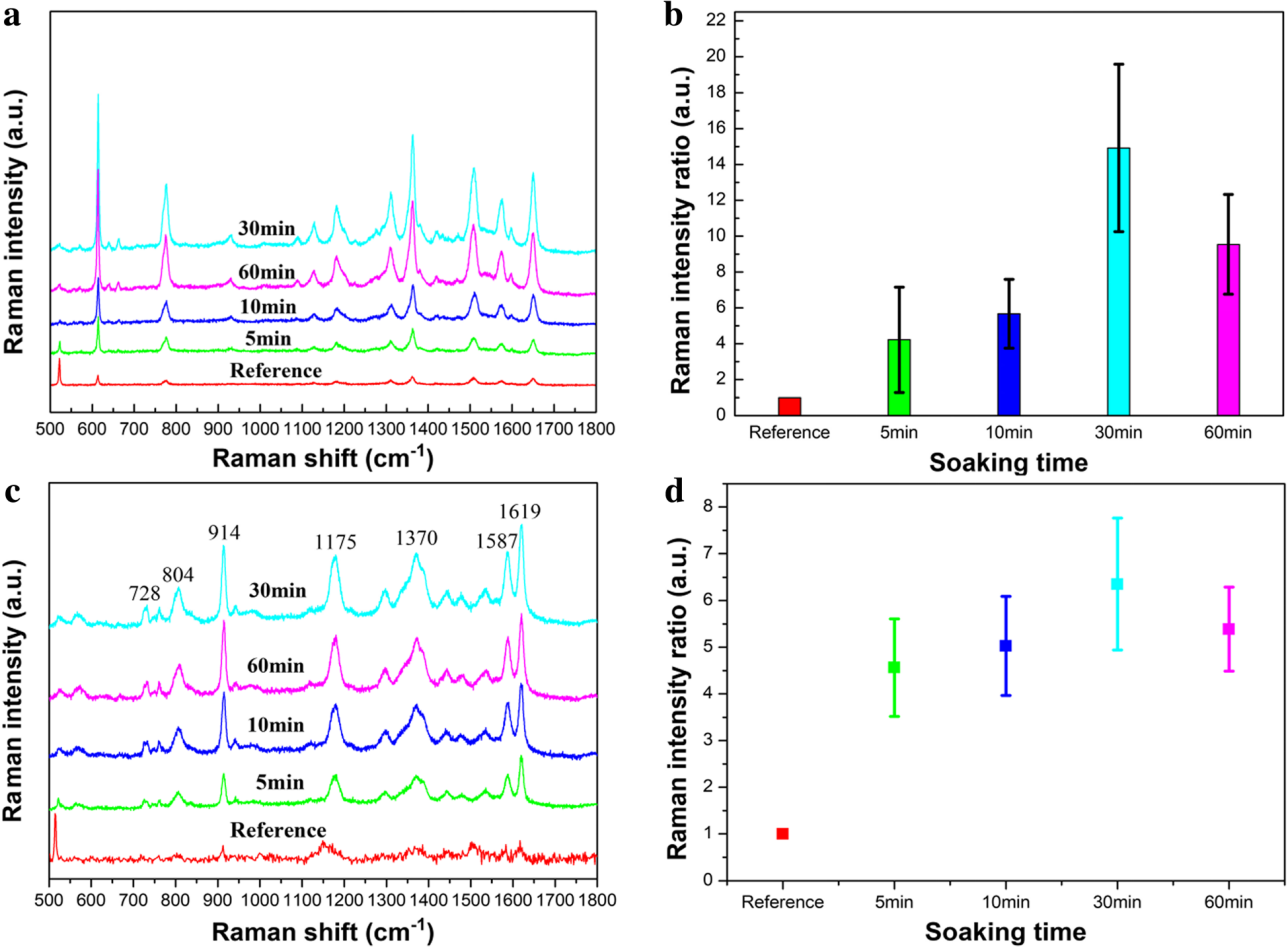
**a** Raman spectra of 10^−7^ M Rh6G on the GNR vertical array substrate with different soaking times. **b** Raman intensity ratio of the peak at 1650 cm^−1^ on the GNR vertical array substrate with different soaking times. **c** Raman spectra of 10^−6^ M CV on the GNR vertical array substrate with different soaking times. **d** Raman intensity ratio of the peak at 1619 cm^−1^ on the GNR vertical array substrate with different soaking times

Interestingly, we initially discover that the Raman intensities of the Rh6G molecules have great changes as the soaking time increases. We performed the tests several times and selected the Raman peak of Rh6G at 1650 cm^−1^ as a reference standard. These obtained results are shown in Fig. 3a and b, which indicates that Raman enhancement effect is optimal with the soaking time of 30 min. We replaced the Rh6G molecules with CV and repeated the experiment. The Raman signals of CV are also given in Fig. 3c and d, which reveals that the tendency of the Raman signals of CV molecules are similar to that of Rh6G molecules for soaking 30 min. Based on this experimental phenomenon, we suspect that the GNR array has collapsed when the substrate is soaked for 60 min and it may be caused by the weakening of electrostatic repulsive force and depletion interaction between nanorods and substrates after the dissolution of CTAB. We used SEM to characterize substrates with different soaking times.

**Fig. 4:**
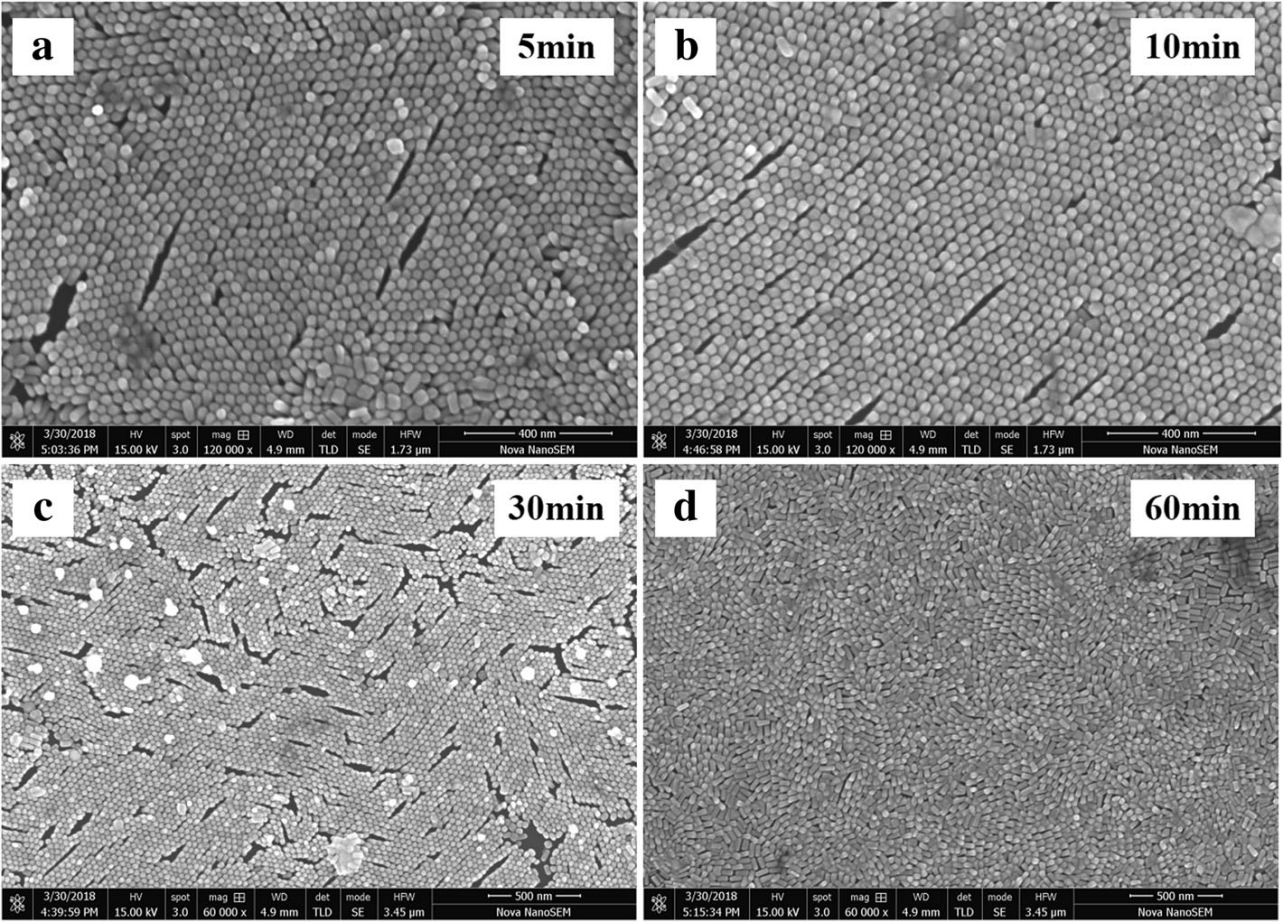
**a**–**d** SEM images of GNR arrays with different soaking times. The soaking time of GNR arrays is 5 min, 10 min, 30 min, and 60 min, respectively

It can be seen from Fig. 4 that the morphology of GNR vertical array hardly changes significantly as the soaking time increases; however, GNR arrays have collapsed and become disordered when the substrate soaking time is 60 min. Based on Fig. 4, the Raman spectrum is explained as follows: During the pre-soaking period, the arrays are relatively stable. The Rh6G molecules adsorbed on the surface of the GNR vertical arrays also increase with the soaking time increases. Under the laser irradiation, the “hot spots” on the surface of the arrays or in the gaps of gold nanorods can enhance the Raman signals of the target molecules. Nevertheless, the intensities of Raman signals of the probe molecules on the disordered substrate are weak due to the number of the “hot spots” between adjacent nanorods is decreasing, in order to better understand the influence of the local electromagnetic field distribution of the GNR vertical arrays on the SERS of the target molecule.

**Fig. 5:**
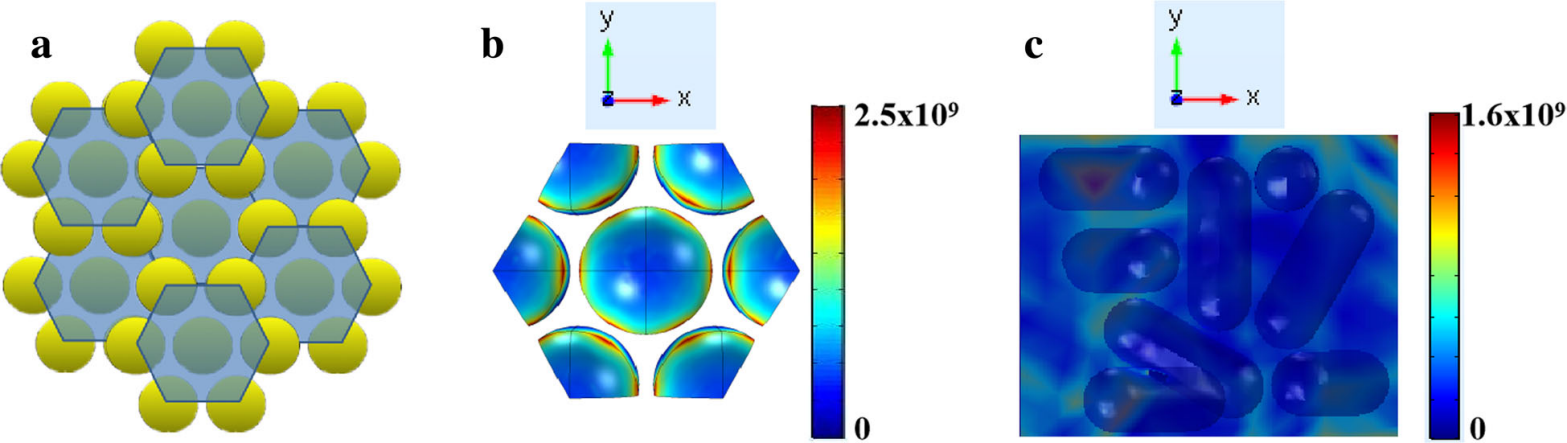
**a** GNR hexagonal array simulation pattern. **b** Local electromagnetic field simulation results of GNR vertical arrays. **c** Local electromagnetic field distribution of disordered GNRs

As shown in Fig. 5, we used FEM to simulate the local electromagnetic field of the substrate under 532 nm laser irradiation. The incident light is circularly polarized and is transmitted along the *z*-axis perpendicular to the *xy* plane. It can be clearly seen from Fig. 5b that the GNR array exhibits an excellent local electromagnetic field enhancement effect compared to the disordered substrate. Based on the electromagnetic field mechanism, the electromagnetic field enhancement SERS formula is given as follows [32]:

1 $$ {\left|{M}_{\mathrm{EM}}\left({\lambda}_{\mathrm{L}},\lambda, {d}_{\mathrm{av}}\right)\right|}^2={\left|\frac{E_{\mathrm{loc}}\left({\lambda}_{\mathrm{L}},{d}_{\mathrm{av}}\right)}{E_{\mathrm{in}}\left({\lambda}_{\mathrm{L}}\right)}\right|}^2\ast {\left|\frac{E_{\mathrm{loc}}\left(\lambda, {d}_{\mathrm{av}}\right)}{E_{\mathrm{in}}\left(\lambda \right)}\right|}^2={\left|{M}_1\left({\lambda}_{\mathrm{L}},{d}_{\mathrm{av}}\right)\right|}^2{\left|{M}_2\left(\lambda, {d}_{\mathrm{av}}\right)\right|}^2 $$

where, |*M*_EM_|^2^is the total electromagnetic field enhancement factor, and |*M*_1_|^2^and |*M*_2_|^2^are the electromagnetic field enhancement factors induced by plasmon resonance coupling and Raman scattering light-plasmon coupling of incident light, respectively. *λ*_L_ and *λ* are the wavelengths of the incident light and the emitted light, respectively. Additionally, *d*_av_ is the average distance from the molecules to the metal surface. *E*_in_ and *E*_loc_ are the intensity of the incident light field and local field. |*M*_EM_|^2^ is roughly proportional to the fourth power of the electric field enhancement without the vector property of the field and the tensor property of the Raman polarization. Therefore, compared with the disordered substrate, the local electromagnetic field around the GNR arrays is relatively strong, and the dense “hot spots” can enhance the SERS activity of the substrate. The result is almost consistent with the experiment of our inference. Thus, in subsequent experiments, all of the GNR array substrates were soaked in the probe molecule solution for 30 min.

**Fig. 6:**
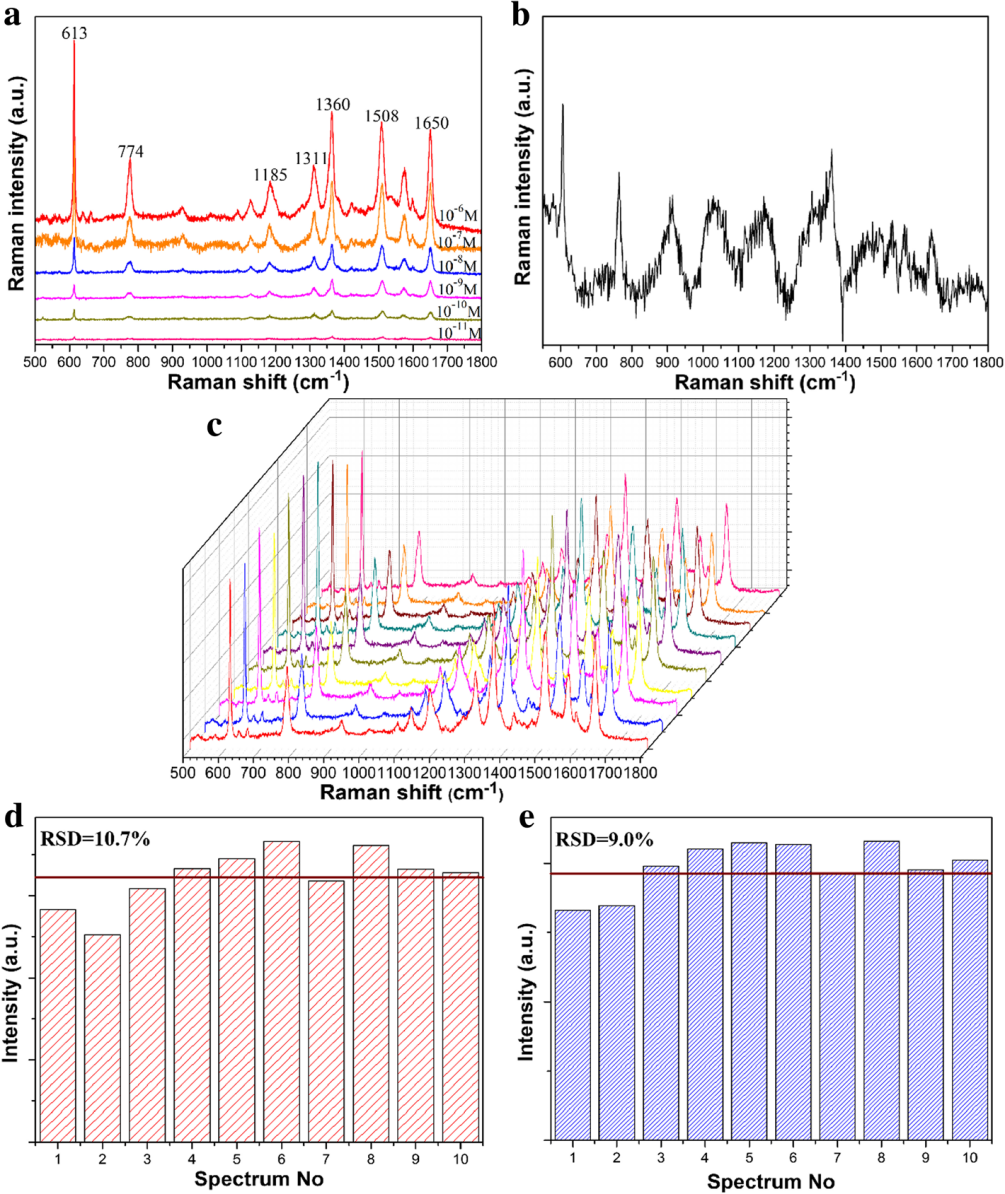
**a** Raman spectra of Rh6G on the GNR vertical array substrate from 10^−6^ to 10^−11^ M, respectively. **b** The Raman spectrum of 10^−3^ M Rh6G on silicon substrate. **c** Raman spectra of Rh6G with a concentration of 10^−7^ M. **d**, **e** Intensity distribution of the peaks at 1360 cm^−1^ and 774 cm^−1^ for the Rh6G with a concentration of 10^−7^ M from 10 different batches of GNR vertical array substrates

In order to effectively evaluate the performance of the substrate-enhanced Raman, we used the Rh6G molecule as the detected target in Raman spectral tests. Based on the above optimal soaking time, the silicon wafer with the GNR vertical arrays was immersed in the probe molecular solution for 30 min. After the soaking, the silicon wafer was rinsed with ethanol and dried. We measure the Raman spectra of the probe molecules with an excitation wavelength of 532 nm. Firstly, the spectra of Rh6G are given in Fig. 6a, which indicates that the Raman signals of Rh6G deposited on the vertical arrays are effectively enhanced. From the range of 500 to 1800 cm^−1^, the Raman peaks at 613 cm^−1^, 774 cm^−1^, 1185 cm^−1^, 1311 cm^−1^, 1360 cm^−1^, 1508 cm^−1^, and 1650 cm^−1^ can be clearly seen, which is consistent with previous reports [33]. The Raman signals of Rh6G decrease as the concentration decreases. The detection sensitivity of the substrate deteriorates when the concentration of Rh6G is adjusted to 10^−11^ M. Now, only these Raman peaks at 613 cm^−1^, 1360 cm^−1^, 1508 cm^−1^, and 1650 cm^−1^ can be observed which indicates that the GNR vertical array substrate presents high sensitivity. Raman scattering signals of target molecules Rh6G are enhanced by the localized electromagnetic field between the gaps of adjacent nanorods. The Raman spectrum of 10^−3^ M Rh6G is also shown in Fig. 6b. Here, we evaluate the enhancement factor (EF) of the SERS substrate [34]:

2 $$ \mathrm{EF}=\frac{{\mathrm{I}}_{\mathrm{SERS}}/{\mathrm{I}}_{\mathrm{Ref}}}{{\mathrm{C}}_{\mathrm{SERS}}/{\mathrm{C}}_{\mathrm{Ref}}} $$

*C*_SERS_ and *C*_Ref_ are the concentration of Rh6G in the SERS substrate (10^−10^ M) and the reference (10^−3^ M), respectively. *I*_SERS_ and *I*_Ref_ are the SERS intensities of GNR arrays after soaking Rh6G and reference Raman signals, respectively. The intensities of the Raman peak at 613 cm^−1^ of the Rh6G are calculated that *I*_SERS_/*I*_Ref_, *C*_SERS_/*C*_Ref_, and the EF are about 0.0965, 10^−7^, and 9.65 × 10^5^, separately. The EF calculated in our experiments is consistent with the magnitude reported in the literature for self-assembled substrate [17, 35, 36].

In general, the substrate has not only good sensitivity but also excellent reproducibility for the SERS applications. In order to present the good reproducibility, we randomly select 10 points from the substrate deposited on Rh6G molecules. As shown in Fig. 6c, Raman peaks of Rh6G are consistent with that of Fig. 6a. Raman peaks of Rh6G at different positions are not moved. Additionally, the relative standard deviation (RSD) of the Raman peak, as an important parameter, is used to evaluate the quality of the substrate reproducibility. Here, relative deviation formula can be expressed as RSD = SD/*I*_m_ [37], where the SD is the standard deviation intensity of the peak and *I*_m_ is the average Raman intensity of the main peak. We calculate the RSD values of the Raman peaks at 1362 cm^−1^ and 774 cm^−1^ from the statistical 10 points, respectively. The RSD values are about 10.7% and 9.0% in Fig. 6d and e, respectively, which indicate that the SERS property of GNR vertical arrays has excellent reproducibility.

**Fig. 7:**
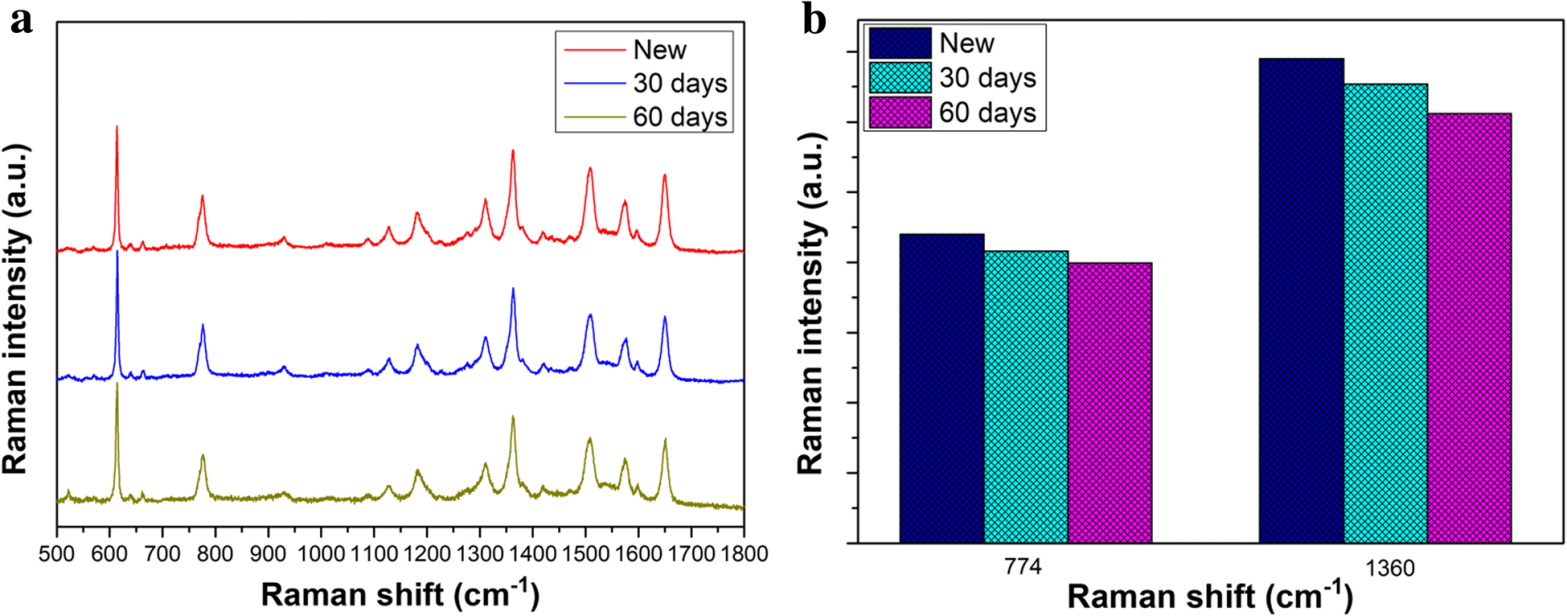
**a** Raman spectra of 10^−7^ M Rh6G on the GNR vertical array substrate with different days. **b** Comparison with the intensities of SERS signals at peaks of 774 cm^−1^ and 1360 cm^−1^

The stability is used as another important factor to evaluate the quality of SERS substrates. In order to verify the substrate with high stability, as shown in Fig. 7a, Raman spectra of Rh6G with the concentration of 10^−7^ M on GNR vertical array substrate are given after 30 days and 60 days. With the time passing by, the SERS signal intensities of Rh6G molecules decrease to some extent after 30 and 60 days because of the loss of SERS activity. However, the Raman signals of the molecules Rh6G on the substrate are not obviously attenuated. The intensities and Raman shift of the characteristic peaks at 774 cm^−1^ and 1360 cm^−1^ are counted in Fig. 6b for the different periods, respectively. Even though the substrate soaked with Rh6G is exposed to air for 60 days, Rh6G on the substrate still maintains a good SERS signal. For the peak at 774 cm^−1^, the loss of Raman signals of Rh6G is about 5.4% and 9.3% after 30 days and 60 days. For the peak at 1360 cm^−1^, the loss of Raman signals of Rh6G is about 5.3% and 11%, respectively. Combined with previous reports [38, 39], it can be considered that the current GNR vertical arrays have good stability. Combine these advantages mentioned above, this substrate possesses great potential in sensing and detection.

## Conclusion

In summary, we have successfully prepared self-assembled GNR vertical arrays by an evaporation method. More importantly, we found that the morphology of GNR vertical arrays can be regulated by changing the soaking time for good Raman enhancement effect. Based on the EM field theory, we used COMSOL software to analyze and discuss the local EM field distribution of GNR vertical array and disorder substrate. The results are almost in agreement with the experiment data. Besides, we studied the SERS activity of the vertical array of GNRs based on the optimal soaking time of the substrate. The as-fabricated substrate can detect Rh6G at concentrations as low as 10^−11^ M due to local electromagnetic field enhancement and show great reproducibility and stability. Therefore, GNR vertical arrays with excellent sensitivity and stability can be used for species detection, sensing, and other fields.

## Abbreviations

CTAB: Cetyltrimethyl ammonium bromide
CV: Crystal violet
FEM: Finite element method
GNRs: Gold nanorods
Rh6G: Rhodamine 6G
RSD: Relative standard deviation
SEF: Surface-enhanced fluorescence
SEM: Scanning electron microscope
SERS: Surface-enhanced Raman scattering
Si: Silicon wafers
SPR: Surface plasmon resonance
